# Endothelial Function and Pro-Inflammatory Cytokines as Prognostic Markers in Acute Coronary Syndromes

**DOI:** 10.3390/diagnostics15081033

**Published:** 2025-04-18

**Authors:** Sotirios Tsalamandris, Leonidas Koliastasis, Antigoni Miliou, Evangelos Oikonomou, Nikos Papageorgiou, Alexis Antonopoulos, George Hatzis, Konstantinos Mourouzis, Georgia Vogiatzi, Gerasimos Siasos, Panagiotis Xaplanteris, Dimitris Tousoulis

**Affiliations:** 1 1st Department of Cardiology, School of Medicine, ‘Hippokration’ General Hospital, National and Kapodistrian University of Athens, 11527 Athens, Greece; stsalamandris@gmail.com (S.T.); lkoliastasis@gmail.com (L.K.); a_miliou@hotmail.com (A.M.); drnpapageorgiou@yahoo.com (N.P.); antonopoulosal@yahoo.gr (A.A.); hatzis_geo@yahoo.gr (G.H.); gvogiatz@yahoo.gr (G.V.); drtousoulis@hotmail.com (D.T.); 2 3rd Department of Cardiology, “Sotiria” Chest Disease Hospital, Medical School, National and Kapodistrian University of Athens, 11527 Athens, Greece; boikono@gmail.com (E.O.); mourouzis2005@yahoo.gr (K.M.); gsiasos@med.uoa.gr (G.S.); 3 Department of Cardiology, Centre Hospitalier Universitaire Saint-Pierre, Université Libre de Bruxelles (ULB), 1000 Brussels, Belgium

**Keywords:** acute coronary syndrome, coronary artery disease, endothelial function, inflammation, pro-inflammatory cytokines

## Abstract

**Background:** Endothelial dysfunction and inflammation are associated with the progression of coronary artery disease (CAD) and the pathophysiology of acute coronary syndrome (ACS). We examined the prognostic role of endothelial function and pro-inflammatory cytokines in patients admitted with ACS. **Methods:** The study population consisted of 864 subjects. From 663 subjects who presented with chest pain, ACS was diagnosed in 460. We additionally recruited 201 consecutive patients with stable CAD. Endothelial function was assessed using flow-mediated dilatation (FMD). Tumor necrosis factor alpha (TNF-α) and interleukin-6 (IL-6) levels were measured via ELISA. Subjects with ACS were followed up for major adverse cardiovascular events (MACE), defined as cardiovascular death, cardiac arrest, myocardial infarction, stroke, nonfatal stroke, other arterial thrombotic events, and hospitalization due to cardiovascular conditions. **Results:** There was a stepwise impairment in FMD, logTNF-α, and logIL-6 in patients with chest pain of non-epicardial CAD etiology compared to patients with stable CAD and those with ACS (*p* < 0.001 for all). Moreover, patients who presented with chest pain had increased odds of ACS in accordance with the increasing levels of TNF-α, IL-6, and impaired FMD (*p* < 0.05 for all). Interestingly, from all these markers, in patients with ACS, we found that only TNF-α levels above 5.19 pg/mL had a 2.5-times-increased risk of MACE compared to patients with TNF-α levels below 5.19 pg/mL, independently of other confounders. **Conclusions:** In the current study, we found that patients who presented with ACS had impaired endothelial function and increased levels of IL-6 and TNF-α.

## 1. Introduction

Arterial atheromatosis is a systemic and progressive disease that starts early in life. Atheromatosis of the coronary arteries may be asymptomatic or may manifest as stable angina or acute coronary syndrome (ACS) [1].

Conventional cardiovascular risk factors (i.e., hypertension, hypercholesterolemia, diabetes, etc.) are implicated in the development of coronary artery disease (CAD), while endothelial dysfunction may be considered as an early stage of the disease [2]. Low-grade systemic inflammation appears not only to impair endothelial function but also to be implicated in the progression of coronary atheromatic lesions [3]. Following the completion of the “Canakinumab Anti-Inflammatory Thrombosis Outcomes Study” (CANTOS) [4], inflammation was further established as a key modifiable factor determining the prognosis and evolution of the disease. Beyond the key role of interleukin (IL)-1β in the progression of atheromatosis, several steps in the pro-inflammatory cascade involve other cytokines, such as IL-6 and tumor necrosis factor alpha (TNF-α) [5,6]. The inhibition of these molecules has been studied as a disease-modifying approach in patients with a myocardial infraction [7].

Despite the ample data linking inflammation and endothelial dysfunction with CAD progression, additional factors underlying plaque rapture and progression from stable CAD to ACS have been identified based on retrospective data, experimental design, post-mortem studies, and epidemiological analysis [8,9].

In the present study, we evaluated the prognostic role in predicting Major Adverse Cardiovascular Events (MACE) of endothelial function and pro-inflammatory cytokines in ACS patients.

## 2. Materials and Methods

### 2.1. Study Population

**Figure 1:**
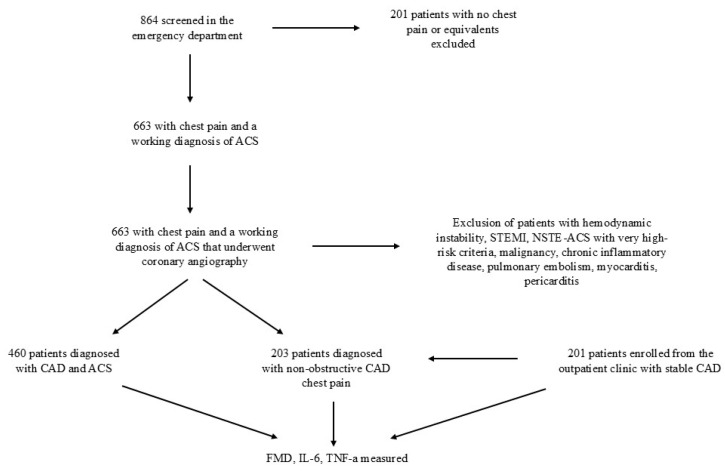
Flowchart of the study. ACS: acute coronary syndrome, CAD: coronary artery disease, IL-6: FMD: flow-mediated dilatation, Interleukin-6, NSTE-ACE: Non-ST-segment elevation acute coronary syndrome, STEMI: ST-segment elevation myocardial infarction, TNF-a: tumor necrosis factor-a.

The study population consisted of 864 subjects, who were screened prospectively from November 2015 until November 2017 from the emergency department and the outpatient clinics of the First Cardiology Department of the Medical School of the National and Kapodistrian University of Athens, Hippokration Hospital, Athens, Greece (Figure 1).

From the total study population, 663 patients presented in the emergency department with chest pain and a working diagnosis of ACS. To attenuate the impact of coronary angiography, percutaneous transluminal angioplasty, and the nitrates use in the examined parameters, we excluded from the study population those with hemodynamic instability, ST-segment elevation myocardial infarction (STEMI), and non-ST segment elevation (NSTE)-ACS patients with very-high-risk criteria (i.e., haemodynamic instability, recurrent chest pain, etc.), according to the recent European Society of Cardiology (ESC) guidelines on the management of patients with NSTE-ACS [10]. Accordingly, all patients underwent a diagnostic coronary angiography. Following the coronary angiography and the established diagnostic algorithm, 460 of these patients were classified as CAD patients and 203 as patients with chest pain of non-CAD etiology. Subjects with comorbidities, such as malignancies, systemic chronic or acute inflammatory diseases, immunological diseases, and pulmonary embolism, were excluded from the study. Subjects who had been diagnosed with myocarditis or pericarditis were also excluded from the study.

In addition, we enrolled 201 consecutive patients from the outpatient clinic of the First Cardiology Department of the Medical School of the National and Kapodistrian University of Athens, Hippokration Hospital, Athens, Greece, with known, stable CAD without any evidence of acute coronary syndrome or unstable angina. We opted to include only patients with epicardial CAD, excluding possible myocardial infarction with non-obstructive coronary artery disease (MINOCA).

Demographics and clinical data were retrieved during the interview and the clinical examination of the patients. We defined “smokers” as current smokers and those who used to smoke at least one cigarette per day and “non-smokers” as those who had never smoked a cigarette in their life or those who had quit smoking at least one year ago. We classified as diabetic those patients who were under insulin or glucose-lowering treatment and those who fulfilled the criteria of the American Diabetes Association for fasting plasma glucose, 2 h plasma glucose during a 75 g oral glucose tolerance test, glycated hemoglobin levels, and random plasma glucose levels [11]. Hypertensive patients were those with systolic blood pressure ≥ 140 mmHg or diastolic blood pressure ≥ 90 mmHg or those who were currently undergoing antihypertensive treatment [12]. The study protocol follows the ethical guidelines set forth in the 1975 Declaration of Helsinki, as confirmed by prior approval from the institution’s human research committee. Furthermore, written informed consent was obtained from all participants.

### 2.2. Study Measurements

#### 2.2.1. Coronary Angiography

Elective coronary angiography was conducted according to local standard procedures, with the operators blinded to the study protocol. Significant coronary artery disease (CAD) was defined as a ≥50% reduction in the luminal diameter of a major epicardial coronary artery, as assessed visually.

#### 2.2.2. Evaluation of Left Ventricle Systolic Performance

All echocardiographic examinations, including two-dimensional and Doppler imaging, were conducted using a General Electric Vivid E9 ultrasound machine (GE Vingmed Ultrasound AS, Horten, Norway) with a 2.5-MHz M5Sc transducer. The left ventricular ejection fraction (EF) was calculated using the Simpson’s biplane method and was used to evaluate the systolic function of the left ventricle 

#### 2.2.3. Evaluation of Endothelial Function

Endothelial function was assessed by measuring flow-mediated dilation (FMD) in the brachial artery, as described in previous studies [13]. For patients presenting with chest pain, all measurements were performed the day after hospital admission in the morning (8.00–10.00 a.m.) and before any invasive procedure. All examinations throughout the study were conducted by the same examiner. FMD results were analyzed by an investigator who was blinded to the sequence of images. Endothelium-independent dilation (EID) was defined as the percentage change in vessel diameter from baseline to the maximum diameter following nitrate administration. The repeatability of the FMD measurement technique at our institution was evaluated using the Bland–Altman method. The repeatability coefficient, calculated using the formula defined by the British Standards Institution (repeatability coefficient = 2 × √(Σdi^2^/N), where N is the sample size and di represents the difference between two paired measurements), was determined to be 5.0%.

#### 2.2.4. Biochemical Measurements

Venous blood samples were centrifuged at 3000 rpm, and the serum/plasma was collected and stored at −80 °C until analysis. For patients presenting with chest pain, blood samples were obtained within 1 h of their arrival at the emergency department. Serum levels of IL-6 and tumor necrosis factor-alpha (TNF-α), markers of systemic and vascular inflammation, were measured using commercially available ELISA kits (BioVendor GmbH, Germany, and R&D Systems, Minneapolis, MN, respectively). Lipid and glucose levels were determined using commercially available enzymatic methods.

#### 2.2.5. Endpoints

After the index event, patients were followed for up to 33 months to monitor the occurrence of major adverse cardiovascular events (MACE). MACE was defined as cardiovascular death, cardiac arrest, myocardial infarction, stroke, other arterial thrombotic events, and hospitalization due to cardiovascular causes.

### 2.3. Statistical Analysis

All variables were assessed for normality using p–p plots. Since the IL-6 and TNF-α values were not normally distributed, they were log-transformed to enhance normality. Normally distributed data are presented as means ± standard deviation, while non-normally distributed data are reported as medians with interquartile ranges. Categorical variables are expressed as valid percentages

Differences between study groups for continuous variables were assessed using one-way analysis of variance (ANOVA). For categorical variables, the chi-square test was used. In the case of significant ANOVA results, post-hoc pairwise comparisons were performed using the Tukey HSD test, with a Bonferroni correction applied to adjust for multiple comparisons. Given the 3 × 2 table structure (six comparisons), the adjusted alpha level was set to αadjusted = 0.056 = 0.0083αadjusted = 60.05 = 0.0083. All reported *p*-values were assessed against this adjusted threshold.

Logistic regression analysis was conducted to evaluate the association between parameters identified as significant in the univariate analysis and the final diagnosis of acute coronary syndrome (ACS) in patients presenting with chest pain. Event analysis for the relationship between biomarkers (FMD, IL-6, TNF-α) and outcomes was performed using the Kaplan–Meier method, with the log-rank test used for group comparisons. Cox proportional hazards analysis was employed to assess the association between significant parameters and the incidence of MACE, adjusting for potential confounders. MACE was defined as cardiovascular death and non-fatal myocardial infarction. Hazard ratios (HR) are presented with 95% confidence intervals (CI). All statistical analyses were conducted using SPSS software, version 18.0 (SPSS Inc., Chicago, IL, USA), and a two-tailed *p*-value < 0.05 was considered statistically significant, except where the Bonferroni correction was applied. A hierarchical approach was followed as a stepwise forward selection method, where variables were added sequentially in a predefined order based on clinical and mechanistic relevance as follows:

Step 1: Included baseline clinical and demographic factors (age, gender, smoking, hypertension, diabetes, hypercholesterolemia, and left ventricular ejection fraction).
Step 2: Added endothelial function (FMD).
Step 3: Included IL-6 to assess systemic inflammation.
Step 4: Added TNF-α to evaluate its unique contribution to cardiovascular events.

## 3. Results

The 864 patients included in the final analysis were followed up for 33 days. Any event fitting the MACE description was catalogued and added to the analysis.

### 3.1. Baseline Characteristics of the Study Population

In subjects with NSTE-ACS, the prevalence of male gender was higher compared to the subjects presenting with chest pain of non-epicardial CAD etiology. Additionally, patients with NSTE-ACS and stable CAD had an increased prevalence of hypertension, diabetes mellitus, and hypercholesterolemia compared to those with chest pain of non-epicardial CAD etiology (Table 1).

### 3.2. Endothelial Dysfunction in Patients with ACS

**Figure 2:**
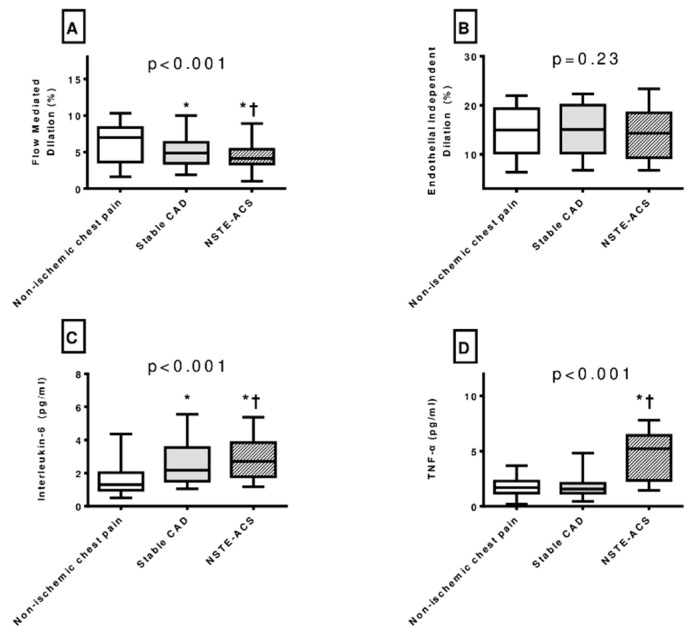
Patients with acute coronary syndrome have increased interleukin 6 and tumor necrosis factor-alpha level as well as impaired endothelial function. (**A**): Impaired flow-mediated dilation in subjects with NSTE-ACS compared to subjects with stable angina and chest pain of non-epicardial CAD etiology. (**B**): Endothelia-independent dilation does not differ in the three study groups (subjects with NSTE-ACS; subjects with stable angina; and subjects with chest pain of non-epicardial CAD etiology). (**C**): Increased interleukin-6 levels in subjects with NSTE-ACS compared to subjects with stable angina and chest pain of non-epicardial CAD etiology. (**D**): Increased tumor necrosis factor-alpha levels in subjects with NSTE-ACS compared to subjects with stable angina and chest pain of non-epicardial CAD etiology. CAD: coronary artery disease; NSTE-ACS: non-ST elevation acute coronary syndrome. Box plots are used to represent the distribution of the examined variables in each study category. *p*-values are based on analysis of variance (ANOVA). Intergroup differences were tested after Scheffe’s correction. *: *p* < 0.05 compared to patients with chest pain of non-epicardial CAD etiology. †: *p* < 0.05 compared to patients with stable CAD.

Interestingly, there was a stepwise impairment in endothelial function in patients with chest pain of non-epicardial CAD etiology compared to patients with stable CAD and those with NSTE-ACS (6.15 ± 2.94% vs. 5.13 ± 2.44% vs. 4.32 ± 2.65%, *p* < 0.001) (Figure 2A, Table 1). There was no difference in EID across the three study groups (Figure 2B, Table 1).

### 3.3. Inflammatory Biomarkers in Patients with ACS

In patients with NSTE-ACS, inflammatory biomarkers (IL-6 and TNF-α) were significantly increased compared to patients with chest pain of non-epicardial CAD etiology and stable CAD patients (Figure 2C,D, Table 1).

### 3.4. Endothelial Dysfunction and Inflammation as Risk Factors for ACS

To examine whether inflammation and endothelial dysfunction are associated with increased risk of ACS in patients who presented to the emergency department with chest pain, we utilize a logistic regression model (Table 2). Accordingly, it was revealed that patients who presented with chest pain had increased odds of NSTE-ACS according to increased levels of TNF-α (OR: 2.47, 95% C.I.: 1.89–3.23, *p* < 0.001) and IL-6 (OR: 3.10, 95% C.I.: 1.92–5.00, *p* < 0.001) and impaired FMD values (OR: 0.84, 95% C.I.: 0.74–0.93, *p* = 0.005).

### 3.5. The Prognostic Role of Endothelial Dysfunction and Inflammation in Patients with ACS

**Figure 3:**
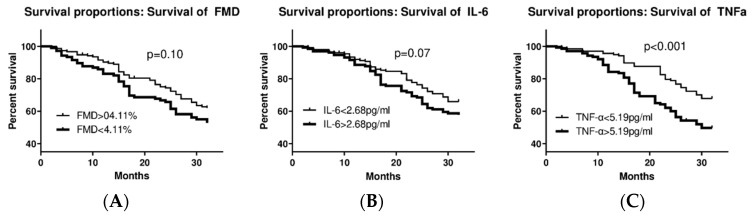
Kaplan–Meier estimates curves for: (**A**) higher FMD (≥4.11%) vs. lower FMD values (<4.11%). (**B**) Higher IL-6 (≥2.68 pg/mL) vs. lower IL-6 levels (<2.68 pg/mL). (**C**) Higher TNF-α (≥5.19 pg/mL) vs. lower TNF-α levels (<5.19 pg/mL). *p*-Values are based on log-rank tests. FMD: flow-mediated dilation; IL-6: interleucin-6; TNF-α: tumor necrosis factor a.

To further examine any possible impact of the examined parameters (inflammatory indices- IL-6, TNF-α-, and endothelial function) on the prognosis of NSTE-ACS patients and the incidence of MACE, Kaplan–Meier estimates were compared between patients with lower binary FMD (<4.11%) vs. upper binary (≥4.11%) (*p* = 0.10) (Figure 3A), lower binary IL-6 (<2.68 pg/mL) vs. upper binary (≥2.68 pg/mL) (*p* = 0.07) (Figure 3B) and lower binary TNF-α (<5.19 pg/mL) vs. upper binary (≥5.19 pg/mL) (*p* < 0.001) (Figure 3C). As shown in Figure 3C, subjects with increased TNF-α levels had significant adverse prognoses.

Since multiple risk factors are implicated in the prognosis of patients with cardiovascular events, we conducted a Cox proportional-hazards analysis of binary TNF-α in patients with successful revascularization after adjustment for age, gender, left ventricular EF, hypertension, diabetes mellitus, hypercholesterolemia, and smoking habits (Table 3). Interestingly, patients with TNF-α levels above 5.19 pg/mL had a 2.5 times increased risk of MACE compared to patients with TNF-α levels below 5.19 pg/mL.

### 3.6. Hierarchical Cox Regression Analysis

To further evaluate the independent and additive prognostic value of endothelial function (FMD) and pro-inflammatory markers (IL-6 and TNF-α), a hierarchical Cox regression analysis was performed. Variables were entered sequentially into the model based on their clinical and mechanistic relevance:

- Step 1: Baseline clinical and demographic factors, including age, gender, smoking, hypertension, diabetes, hypercholesterolemia, and the left ventricular ejection fraction (LVEF).
- Step 2: Addition of endothelial function as measured by flow-mediated dilation (FMD).
- Step 3: Inclusion of IL-6 to assess systemic inflammation.
- Step 4: Inclusion of TNF-α to evaluate its unique contribution to adverse cardiovascular events.

The results are summarized in Table 4. TNF-α significantly improved the predictive ability of the model, with a hazard ratio (HR) of 2.5 (95% CI: 1.11–6.34; *p* = 0.01) for MACE in patients with TNF-α ≥ 5.19 pg/mL, even after adjusting for endothelial dysfunction and IL-6. This finding highlights the critical role of TNF-α in the prognosis of patients with ACS.

The hierarchical analysis demonstrates that TNF-α retains its prognostic value for predicting MACE after accounting for endothelial dysfunction (FMD) and systemic inflammation (IL-6). The additive model improvements were statistically significant at each step, as confirmed by the likelihood ratio test.

## 4. Discussion

In the present study, we examined the levels of pro-inflammatory molecules and the endothelial function status in subjects according to CAD evolution (NSTE-ACS, stable CAD, chest pain of non-epicardial CAD etiology). We demonstrated that patients with CAD have increased levels of pro-inflammatory molecules (IL-6, TNF-α) and significant endothelial impairment compared to subjects with no evidence of CAD. Importantly, we found that even after adjustment for classic risk factors and potential confounders, systemic inflammation, and endothelial dysfunction are common findings in subjects presenting with NSTE-ACS, emphasizing the underlying role of endothelial impairment and inflammation in plaque vulnerability and in the progression of stable CAD to NSTE-ACS. In addition, systemic inflammation at presentation may determine adverse long-term prognosis in the context of NSTE-ACS.

### 4.1. Endothelial Function in Acute Coronary Syndromes

The endothelium plays a key role in vascular homeostasis. Nitric oxide (NO) is produced by a healthy endothelium and maintains vascular homeostasis, providing vasodilation and protection of the vessel wall from injuries, regulating the regeneration of the damaged endothelium and smooth muscle cell growth. Endothelial dysfunction is not only considered as an early manifestation of arterial atheromatosis [2,14], but it is also exposed to the impact of several cardiovascular risk factors. Importantly, recent studies have reported that, within the coronary network, endothelial dysfunction progresses gradually from the microvascular level to the large epicardial arteries [14].

ACSs are common following the rapture or the erosion of the fibrous cap of a plaque in the large epicardial arteries [15]. Endothelial dysfunction has been associated with the prognosis of patients with ACS and this may be related to oxidative damage, activation of the thrombosis fibrinolysis system, or circulating endothelial microparticles following ACS [16,17]. Treatment with statins improves endothelial function and, when they are used after an ACS, they have been associated with beneficial outcomes.

Moreover, inflammation within coronary plaque drives ACSs. Specifically, interleukin-1 and TNF-α have an established role in atherogenesis and the vessel response to injury, and they have already been used as a therapy target in several studies [18,19,20]. Interestingly, in numerous studies, inflammation consistently demonstrates an additive prognostic value to the established biomarkers, such as cTn levels, in ACS patients [20,21]. Furthermore, the anti-inflammatory effect of statins has been shown to be of particular importance, as these agents can actually ameliorate CAD prognosis [22].

However, no definitive data exist on the impact of endothelial dysfunction per se on plaque rapture. High values of shear stress on vessel walls promote the development of atheromatic plaques with high-risk characteristics, as well as the impairment of endothelial cell function that promotes the progression of the atherosclerotic lipid core [23].

In our study, we revealed not only that endothelial function is impaired in subjects with ACS compared to subjects with stable CAD but also that, in patients with chest pain when endothelial dysfunction is present, it increases the odds of significant CAD. The above finding provides possible clues on the conditions that precede plaque rapture and thus suggest potential new treatment targets.

### 4.2. Inflammatory Substrate in Acute Coronary Syndromes

There is a good deal of evidence for the close relationship between inflammatory stimuli and ACS. From an epidemiologic perspective, community-acquired infections have been associated with increased incidence of ACS, which may be provoked by the direct impact of adhesion molecules (i.e., vascular cell adhesion molecule-1; soluble intercellular adhesion molecule-1) and inflammatory cytokines (i.e., IL-6) on the fibrous cap [8,24]. A few years ago, thermal heterogeneity in coronary plaques was established as a risk factor for ACS [25]. Recently, positron emission tomography with computed tomography (PET-CT), using specific tracers targeting inflammatory lesions (18 F-sodium fluoride), has revealed an ability to identify high-risk coronary atheromatic plaques [26]. In the light of the positive results of “Canakinumab Anti-Inflammatory Thrombosis Outcomes Study” (CANTOS) [4]. This was the first study that documented a positive outcome in patients with myocardial infarction by targeting specific steps in the inflammatory cascade. The concept of inflammation as a trigger factor of ACS has emerged to the forefront in terms of research interest. Indeed, targeting specific inflammatory molecules during the acute phase or at the evolution of myocardial infarction may represent an additive treatment to the current state-of-the-art management of patients with ACS. JAK-2 mutations, clinically manifesting as polycythemia vera, essential thrombocythemia, or primary myelofibrosis, carry an increased thrombotic risk, potentially leading to ACS and coronary ischemia [27,28].

The spectrum of ACS includes not only epicardial CAD but also non-obstructive coronary artery disease. The diagnostic algorithm includes the addition of magnetic resonance imaging, to find out the exact cause and pathophysiology [29]. It is possible that endothelial dysfunction and inflammation play a significant role in the pathophysiology of MINOCA, but this was beyond the scope of this study [30].

In the present study, we confirmed that circulating levels of IL-6 and TNF-α are increased in patients with ACS concurrently with endothelial dysfunction, confirming not only the inflammatory theory of vulnerable plaque but also providing possible additional targets for therapy and the molecule-specific modification of the inflammatory cascade in the acute settings.

### 4.3. Limitations

A notable limitation of this study is the absence of cardiac magnetic resonance imaging (MRI) for patients presenting with acute coronary syndrome (ACS) and non-obstructive coronary arteries (NOCA). Cardiac MRI is increasingly recognized as an essential diagnostic modality in such cases, particularly for myocardial infarction with non-obstructive coronary arteries (MINOCA). Early MRI evaluation can detect ischemic injury through late gadolinium enhancement, aiding in distinguishing ischemic etiologies, such as myocardial infarction, from non-ischemic causes such as myocarditis or Takotsubo cardiomyopathy. Tamis-Holland et al. emphasize the role of MRI in standardizing the diagnostic approach for MINOCA, while Scally et al. illustrate its ability to identify myocardial abnormalities that might otherwise remain undetected [27,28]. By uncovering microvascular dysfunction or inflammatory processes, cardiac MRI could have provided deeper insights into the ischemic mechanisms in this cohort. Future studies should consider integrating cardiac MRI to refine the diagnostic and therapeutic strategies for ACS patients with NOCA. Additional inflammatory markers such as C-reactive protein and the Neutrophil/Lymphocyte ratio could have been included as they are low-cost measures with possible clinical implications [31].

## 5. Conclusions

This study demonstrates significant endothelial dysfunction and elevated levels of inflammatory cytokines, particularly IL-6 and TNF-α, in patients with acute coronary syndrome (ACS) compared to those with stable coronary artery disease (CAD) or chest pain of non-epicardial CAD ischemic origin. Among these markers, elevated TNF-α levels may serve as a potential prognostic indicator for predicting major adverse cardiovascular events (MACE) in patients with non-ST elevation ACS (NSTE-ACS). While these findings suggest that TNF-α could play a role in cardiovascular risk stratification, the observational nature of the study and the lack of advanced diagnostic imaging techniques, such as cardiac MRI, limit our ability to draw definitive causal conclusions. Future studies incorporating these methodologies are needed to validate our findings and further elucidate the mechanisms linking endothelial dysfunction and systemic inflammation to cardiovascular outcomes.

## Figures and Tables

**Table 1:** Baseline demographic and clinical characteristics of the patient population.

	Patients with Chest Pain of Non-Epicardial CAD Etiology	Patients with Stable CAD	Patients with NSTE-ACS	Total	*p* Value
Number of subjects	203	201	460	864	
Age (years)	60 ± 13	61 ± 11	62 ± 11	N/A	0.25
Male sex (%)	59	69	71	199	0.003
BMI (kg/m^2^)	27.47 ± 3.58	27.82 ± 3.87	27.94 ± 3.38	N/A	0.18
EF (%)	57 ± 4	51 ± 7 *	50 ± 7 *	N/A	<0.001
Current smokers (%)	30	21	26	77	0.11
Hypertension (%)	46	77	74	197	<0.001
Diabetes mellitus (%)	19	28	29	76	0.05
Hypercholesterolemia (%)	54	71	71	196	<0.001
FMD (%)	6.15 ± 2.94	5.13 ± 2.44 *	4.32 ± 2.65 *†	N/A	<0.001
EED (%)	14.98 ± 4.67	15.04 ± 4.78	14.28 ± 5.29	N/A	0.23
IL-6 (pg/mL)	1.31 (0.97, 2.02)	2.28 (1.53, 3.87) *	2.70 (1.77, 3.84) *†	N/A	<0.001
TNF-α (pg/mL)	1.90 (1.34, 2.34)	1.65 (1.31, 2.28)	5.20 (2.34, 6.42) *†	N/A	<0.001

*: *p* < 0.05 compared to patients with chest pain of non-epicardial CAD etiology. †: *p* < 0.05 compared to patients with stable CAD. BMI: body mass index; EF: ejection fraction; FMD: flow-mediated dilation; EED: endothelial independent dilation; IL-6: interleukin-6; TNF-α: tumor necrosis factor-alpha; CAD: coronary artery disease; NSTE-ACS: non-ST elevation acute coronary syndrome.

**Table 2:** Logistic regression analysis for the evolution of ACS in patients presenting with chest pain.

	Odds Ratio	95% Confidence Interval	*p*-Value
Age (years)	1.01	0.99, 1.04	0.36
Male sex	3.81	1.67, 8.71	0.001
EF (%)	0.76	0.71, 0.82	<0.001
Hypertension	1.35	0.63, 2.91	0.43
Diabetes mellitus	1.01	0.41, 2.45	0.98
Hypercholesterolemia	1.41	0.68, 2.92	0.35
FMD (%)	0.84	0.74, 0.94	0.005
IL-6 (pg/mL)	3.10	1.92, 5.00	<0.001
TNF-α (pg/mL)	2.47	1.89, 3.23	<0.001

For sex, the reference category is female; for hypertension, diabetes mellitus, and hypercholesterolemia, the reference category is the negative status. EF: ejection fraction; FMD: flow mediated dilation; IL-6: interleukin-6; TNF-α: tumor necrosis factor-alpha.

**Table 3:** Cox regression analysis for the risk of cardiovascular events.

	Hazard Ratio	95% Confidence Interval	*p*-Value
Age (years)	0.99	0.95, 1.03	0.38
Sex	0.67	0.32, 1.34	0.14
LVEF (%)	1.01	0.95, 1.06	0.74
Hypertension	0.94	0.34, 2.61	0.67
Diabetes mellitus	0.66	0.23, 1.92	0.31
Hypercholesterolemia	0.59	0.26, 1.33	0.21
Smoking	1.11	0.457, 2.68	0.56
TNF-α			0.01
<5.19 pg/mL	-	-	
≥5.19 pg/mL)	2.50	1.11, 6.34	

For sex, the reference category is female; for hypertension, diabetes mellitus, hypercholesterolemia, and smoking the reference category is the negative status. LVEF: left ventricular ejection fraction; TNF-α: tumor necrosis factor-alpha.

**Table 4:** Hierarchical Cox regression analysis for the risk of major adverse cardiovascular events (MACE).

Step	Variables Included	Hazard Ratio (HR)	95% Confidence Interval	*p*-Value
1	Clinical factors (age, gender, smoking, hypertension, diabetes, hypercholesterolemia, LVEF)	1.01	0.95–1.06	0.38
2	Endothelial function (FMD)	1.15	1.05–1.27	0.005
3	IL-6	1.78	1.30–2.43	<0.001
4	TNF-α	2.50	1.11–6.34	0.01

LVEF: left ventricular ejection fraction; FMD: flow-mediated dilation; IL-6: interleukin-6; TNF-α: tumor necrosis factor-alpha.

## Data Availability

The original contributions presented in the study are included in the article; further inquiries can be directed to the corresponding author.

## Abbreviations

ACS	Acute coronary syndrome
CAD	Coronary artery disease
CANTOS	Canakinumab Aniinflammatory Thrombosis Outcome Study
EID	Endothelium independent dilatation
ESC	European society of Cardiology
FMD	Flow-mediated dilatation
IL	Interleukin
MACE	Major adverse cardiovascular events
MINOCA	Myocardial infarction with non-obstructive coronary artery disease
NSTE	Non-ST segment elevation
STEMI	ST-segment elevation myocardial infarction
TNF	Tumor necrosis factor
